# Post-COVID depression among a sample of Egyptian patients and its associated factors

**DOI:** 10.1186/s43168-021-00086-7

**Published:** 2021-10-11

**Authors:** Hieba Gamal Ezzelregal, Azza Mohammed Hassan, Rehab Serag Mohamed, Noha Othman Ahmed

**Affiliations:** 1 Chest Department, Ain Shams University, 3 Ali Elabbady street, Ain shams, Cairo, Egypt; 2 Community, Environmental and Occupational Medicine Department, Ain Shams University, Cairo, Egypt; 3 Neuropsychiatry Department, Faculty of Medicine, Ain Shams University, Cairo, Egypt

**Keywords:** COVID-19, Depression, Psychiatry, Pulmonary disease, Psychological dyspnoea

## Abstract

**Background:**

Depression is classified as a mood disorder. It may be described as feelings of sadness, loss, or anger that interfere with a person’s everyday activities. Nowadays, we are in COVID-19 pandemic. From practice after COVID-19 illness resolves, some of the recovering patients return back smoothly to their pre-illness life. Others experience different mood changes. Anxiety and depression are the most common. Those patients with improving general health, radiology, and oxygenation have different somatic complaints such as sensation of dyspnoea. Psychological support and psychiatric evaluation can help them to overcome this situation and get rid of dyspnoea sensation. This work aimed to evaluate the relation between COVID-19 survivors and depression and to how extent this could affect functional status of the study participants.

**Results:**

This work recruited 102 adult patients as a sample of Egyptians who were positive PCR for SARS-COV2, turned negative and free of symptoms for 1 month or more which include physicians, nurses, employees, and literate health care workers of Ain Shams University hospitals attending chest outpatient clinic for follow-up. The majority were 47.1% in age group (35–55 years), sixty two (60.8%) participants were females, 74.5% had high education, and 24.5% were smokers. The most frequent symptom reported by study participants as the most annoying COVID-19 symptom was fever (32.4%). Beck depression inventory score showed that 59 (57.8%) participants had no depression, 24 (23.5%) had mild depression, 16 (15.7%) had moderate depression, and only 3 (2.9%) participants had severe depression. Logistic regression analysis was done to measure effect of steroid use and grade of dyspnoea on development of moderate or severe post-COVID depression and showed that higher grades of dyspnoea were associated with higher probability of development of moderate or severe post-COVID depression (*p* value < 0.05).

**Conclusion:**

As predicted, COVID-19 survivors presented a high prevalence of psychiatric sequelae. Age, sex, and education level were important association factors. Higher educational level was associated with higher score of depression due to increased awareness of the current pandemic issue. Steroids’ use was proposed as a cause of depression since the majority of moderate or severe depression group were on steroids. Higher grades of dyspnoea were associated with higher probability of development of moderate or severe post-COVID depression. It is suggested that COVID-19 survivors should be assessed, to properly diagnose and treat any psychiatric conditions, to reduce the disease burden.

## Background

Coronavirus is one of the major pathogens that primarily targets the human respiratory system. Previous outbreaks of coronaviruses (CoVs) include the severe acute respiratory syndrome (SARS)-CoV and the Middle East respiratory syndrome (MERS)-CoV which have been previously characterized as agents that are a great public health threat. Nowadays we are in COVID-19 pandemic [1].

Depression is classified as a mood disorder. It may be described as feelings of sadness, loss, or anger that interfere with a person’s everyday activities. Sad and upsetting events happen to everyone. But, if you are feeling down or hopeless on a regular basis, you could be dealing with depression [2]. People may also have a lot of concerns around school or work, their finances, their ability to take part in important community and social events and hobbies, and other important parts of their lives [3]. Quarantine time alone is a major factor in post-COVID depression.

From practice after COVID-19 illness resolves, some of the recovering patients return back smoothly to their pre-illness life. Others experience different mood changes. Anxiety and depression are the most common. Those patients with improving general health, radiology, and oxygenation have different somatic complaints such as sensation of dyspnoea. Only psychological support and psychiatric evaluation can help them to overcome this situation and get rid of dyspnoea sensation. So this work measured the frequency of depression among a sample of post-COVID-positive PCR patients who turned negative by PCR, assessed the degree of dyspnoea after being asymptomatic, and measured the relation between depression and dyspnoea score.

## Methods

- This was a descriptive cross-sectional study, recruited 102 participants from Ain Shams University Hospitals’ medical staff (physicians, nurses) and employees of the Ain Shams University who were attending the separate COVID outpatient clinic for diagnosis or follow-up. Using Epi Info 7 program for sample size calculation and assuming that 50% of study participants suffering from post-COVID depression, with margin of error = 10% and at 95% confidence level, sample size of at least 100 participants was needed.
- *Exclusion criteria*: Patients with breathing difficulty (≥ 30 breaths per minute), resting oxygen saturation ≤ 92% on room air or need for domiciliary oxygen, any abnormality in CT chest, severe complications or need for hospitalization, patients already on anti-depressant or anti-anxiety medication, suffering from mania, co-morbid conditions like uncontrolled diabetes, uncontrolled hypertension, chronic liver disease, malignancy, HIV/AIDS, and chronic renal disease undergoing regular dialysis were excluded.
- *All enrolled participants were subjected to:* (after obtaining informed consent from them and informing them about the purpose of the work):

1. Full history taking age, gender, smoking, education, illness duration, comorbidities, and the most distressing symptoms
2. Time being oxygen saturation, HRCT Chest
3. Beck depression inventory, BDI-II [4] (Arabic version) [5]: It is a validated and reliable tool. The original BDI was first published in 1961.The BDI-II is a 1996 revision of the BDI, developed in response to the American Psychiatric Association’s publication of the Diagnostic and Statistical Manual of Mental Disorders, 4th Edition, which changed most of the diagnostic criteria for Major Depressive Disorder.

The BDI-II is a self-report, multiple choice inventory. It contains 21 questions, each answer being scored on a scale value of 0 to 3 based on the severity of each item. Higher total scores indicate more severe depressive symptoms. It is a widely used indicator of the severity of depression.

0–13 = minimal range

14–19 = mild depression

20–28 = moderate depression

29–63 = severe depression.

4) Modified Medical Research Council (mMRC) dyspnoea score [6]:

**Table:** 

Grade	Description of Breathlessness
Grade 0	I only get breathless with strenuous exercise
Grade 1	I get short of breath when hurrying on level ground or walking up a slight hill
Grade 2	On level ground, I walk slower than people of the same age because of breathlessness, or I have to stop for breath when walking at my own pace on the level
Grade 3	I stop for breath after walking about 100 yards or after a few minutes on level ground
Grade 4	I am too breathless to leave the house or I am breathless when dressing

These data and questionnaires were fulfilled by all participant then analysed.

### Ethical consideration

All the study steps were consistent with the ethical principles of Declaration of Helsinki for medical research involving human subjects and were approved by the Faculty of Medicine, Ain Shams University FMASU R 06 / 2020/ 2021

### Statistical data analysis

Data were analysed using computer program SPSS (Statistical Package for the Social Science; SPSS Inc., Chicago, IL, USA) release 15 for Microsoft Window (2006). Data were presented as number and percentage. Chi-square test (or Fisher’s exact test) was used to compare data between different groups. Logistic regression analysis was done to measure relation between different participants’ characteristics and presence of moderate or high levels of depression. *P* value < 0.05 was considered statistically significant.

## Results

This was a descriptive cross-sectional study which recruited 102 persons. 47.1% were in age group (35–55 years), 40.2% in age group (20–34 years), and 12.7% were above 55 years old. Sixty-two (60.8%) participants were females, 74.5% had high education, and 24.5% were smokers. The most frequent symptom reported by study participants as the most annoying COVID-19 symptom was fever (32.4%) followed by body pains (20.6%). Fifty-six (54.9%) participants had co-morbid conditions and 53.9% of them were using steroids. Fifty-one (50%) participants had O_2_ saturation level above 95% and 45.1% had grade 2 dyspnoea (Table 1).

**Table 1:** Characteristics of the study participants

		***N***	Percent
**Age (years)**	**20–34**	41	40.2
	**35–55**	48	47.1
	**More than 55**	13	12.7
**Gender**	**Male**	40	39.2
	**Female**	62	60.8
**Education level**	**High education**	76	74.5
	**Secondary education**	22	21.6
	**Below secondary education**	4	3.9
**Smoking**	**Yes**	25	24.5
	**No**	77	75.5
**The most annoying symptom**	**Fever**	33	32.4
	**Body pains**	21	20.6
	**Diarrhoea**	3	2.9
	**Loss of smell**	8	7.8
	**Loss of taste**	1	1.0
	**Dyspnoea**	18	17.6
	**Cough**	18	17.6
**Co-morbidities**	**Asthma**	9	8.8
	**COPD**	3	2.9
	**HTN**	2	2.0
	**DM**	7	6.9
	**Renal disease**	8	7.8
	**Cardiac disease**	11	10.8
	**Others**	16	15.7
	**No co-morbidity**	46	45.1
**Steroid use**	**Yes**	55	53.9
	**No**	47	46.1
**O2 saturation**	**92, 93%**	20	19.6
	**94, 95%**	31	30.4
	**More than 95%**	51	50.0
**mMRC dyspnoea score**	**Grade 1**	34	33.3
	**Grade 2**	46	45.1
	**Grade 3**	11	10.8
	**Grade 4**	11	10.8

*mMRC* modified Medical Research Council, *DM* diabetes mellitus, *HTN* hypertension, *COPD* chronic obstructive pulmonary disease

**Fig. 1:**
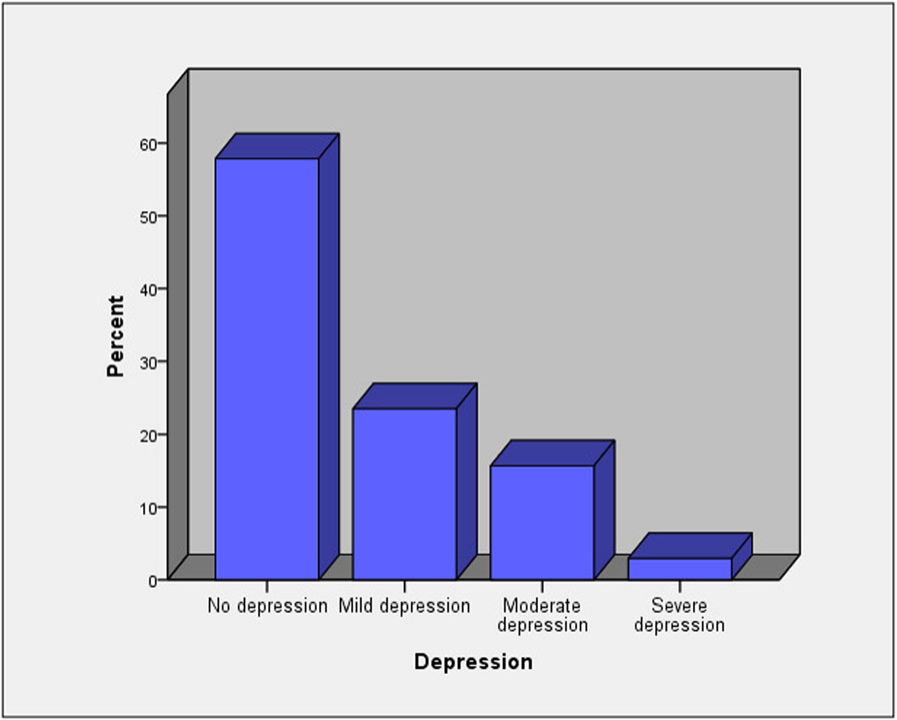
Frequency of post-COVID depression among study participants

Regarding frequency of post-COVID depression among study participants, Beck depression inventory score showed that 59 (57.8%) participants had no depression, 24 (23.5%) had mild depression, 16 (15.7%) had moderate depression, and only 3 (2.9%) participants had severe depression (Fig. 1).

Group of participants with moderate or severe depression (19 persons) were compared with no or mild depression group (83 persons) regarding personal characteristics. No significant difference was found between two groups except for gender (68.4% of moderate or severe depression group were males and 67.5% of no or mild depression group were females) (*P* value < 0.01), education level 47.4% of moderate or severe depression group received higher education) (*p* value < 0.01), steroid use (89.5% of moderate or severe depression group were using steroids compared to only 45.8% of no or mild depression group) (*p* value < 0.01), and dyspnoea score (84.2% of moderate or severe depression group hade grade 2 dyspnoea compared to 36.1% of no or mild depression group) (*p* value < 0.01) (Table 2).

**Table 2:** Factors associated with post-COVID depression

		Post-COVID depression				***P*** value
		No or mild depression (***N***=83)		Moderate or severe depression (***N***=19)		
		***N***	Percent	***N***	Percent	
**Age (years)**	**20–34**	37	44.6	4	21.1	0.06
	**35–55**	38	45.8	10	52.6	
	**More than 55**	8	9.6	5	26.3	
**Gender**	**Male**	27	32.5	13	68.4	0.004
	**Female**	56	67.5	6	31.6	
**Education level**	**High education**	67	80.7	9	47.4	<0.001
	**Secondary education**	16	19.3	6	31.6	
	**Below secondary education**	0	0.0	4	21.1	
**Smoking**	**Yes**	22	26.5	3	15.8	0.33
	**No**	61	73.5	16	84.2	
**The most annoying symptom**	**Fever**	25	30.1	8	42.1	0.38
	**Body pains**	16	19.3	5	26.3	
	**Diarrhoea**	2	2.4	1	5.3	
	**Loss of smell**	8	9.6	0	0.0	
	**Loss of taste**	1	1.2	0	0.0	
	**Dyspnoea**	17	20.5	1	5.3	
	**Cough**	14	16.9	4	21.1	
**Co-morbidities**	**Yes**	47	56.6	9	47.4	0.46
	**No**	36	43.4	10	52.6	
**Steroid use**	**Yes**	38	45.8	17	89.5	0.001
	**No**	45	54.2	2	10.5	
**O**_**2**_ **saturation**	**92, 93**	15	18.1	5	26.3	0.20
	**94, 95**	23	27.7	8	42.1	
	**More than 95**	45	54.2	6	31.6	
**mMRC dyspnoea score**	**Grade 1**	33	39.8	1	5.3	0.001
	**Grade 2**	30	36.1	16	84.2	
	**Grade 3**	11	13.3	0	0.0	
	**Grade 4**	9	10.8	2	10.5	

*mMRC* modified Medical Research Council

Logistic regression analysis was done to measure effect of steroid use and grade of dyspnoea on development of moderate or severe post-COVID depression and showed that higher grades of dyspnoea were associated with higher probability of development of moderate or severe post-COVID depression (*p* value < 0.05) (Table 3).

**Table 3:** Logistic regression analysis for effect of steroid use and grade of dyspnoea on development of moderate or severe post-COVID depression

	***B***	Sign.	Odds ratio	95% C.I. for odds ratio	
				Lower	Upper
**Steroid use**	0.88	0.06	2.41	0.96	6.06
**Grade 2 mMRC**	1.13	0.03	3.10	1.13	8.48
**Grade 3 mMRC**	1.90	0.01	6.70	1.52	29.48
**Grade 4 mMRC**	2.11	0.01	8.20	1.78	37.84

*mMRC* modified Medical Research Council

Reference group was grade 1 mMRC

## Discussion

Depression is classified as a mood disorder. It may be described as feelings of sadness, loss, or anger that interfere with a person’s everyday activities. Sad and upsetting events happen to everyone. But, if you’re feeling down or hopeless on a regular basis, you could be dealing with depression (2). People may also have a lot of concerns around school or work, their finances, their ability to take part in important community and social events and hobbies, and other important parts of their lives(3). Quarantine time alone is a major factor in post-COVID depression.

Psychopathology and psychiatric sequelae were noted after previous coronavirus outbreaks and were induced as infection-triggered disorder of the immune system. The pandemic of severe acute respiratory syndrome coronavirus (COVID-19) could be linked to psychiatric modulations. In this work, the depression impact of COVID-19 in survivors without complications was investigated on patients’ dyspnoea.

Depression symptoms were screened in 102 adults surviving COVID-19 by using the Beck depression inventory, validated BDI-II in Arabic. Clinical and demographic characteristics of the patients are put into consideration as an important influence in the grading of depression sequelae of COVID-19. Sixty-two (60.8%) participants were females, 74.5% had high education, and 24.5% were smokers. The most frequent symptom reported by study participants as the most annoying COVID-19 symptom was fever (32.4%) followed by body pains (20.6%). Fifty-six (54.9%) participants had co-morbid conditions and 53.9% of them were using steroids. Fifty-one (50%) participants had O2 saturation level above 95% and 45.1% had grade 2 dyspnoea.

Psychiatric consequences to SARS-CoV-2 infection can be caused both by the immune response to the virus itself or by psychological stressors such as social isolation, psychological impact of a novel severe and potentially fatal illness, concerns about infecting others, and stigma. The immune response to coronaviruses induces local and systemic production of cytokines, chemokines, and other inflammatory mediators [7].

Neuroinflammation, blood-brain-barrier disruption, peripheral immune cell invasion into the CNS, neurotransmission impairment, hypothalamic-pituitary adrenal (HPA) axis dysfunction, microglia activation, and indoleamine 2,3-dioxygenase (IDO) induction, all represent interaction pathways between immune systems and psychopathological mechanism underpinning psychiatric disorders [8, 9].

Furer et al. found that nearly ½ of the participants had depressive symptoms indicating that the uncertainty of the epidemic progression would cause greater psychological pressure on the public. The possible reason for these mental problems may be related to the “hypochondriac concerns” (worry about being infected) and feared that the epidemic is hard to control [10].

In agreement with previous studies, younger patients showed higher levels of depression with 52.6% of cases with age group (35–55 years) suffered moderate or severe depression in comparison to 26.3% of age group above 55 years old. This describes a worse psychological impact of COVID-19 pandemic in younger age group. Although the reasons for this are complex, it may be due to that this segment of population were most exposed to news from social media, which is usually overwhelmed with fake news, unconfirmed information, and rumours [11–13].

In agreement with previous study [14] and in contrast to other [15], men in the current study suffered from depression at greater levels than women during the COVID-19 outbreak, which was different from previous research that women were more likely to have depression than men due to their sensitive emotions. The occupation and the awareness of the female health care workers might be the cause of their less depression symptoms.

Neither oxygen saturation level at follow-up nor most annoying symptoms were significantly associated with depression suggesting that psychiatric illness was not a manifestation of physical symptoms which comes hand in hand with Mazza et al. [16].

This work studies a sample of Egyptians including post-COVID-19 adult physicians, nurses, and employees, literate health care workers of the Ain Shams University older than 18 years old. During the viral epidemics, the mental health of those HCWs confronts serious challenges [17]. HCWs face the death of their colleagues and threats to their lives. Moreover, the fear of becoming infected, the absence of an effective social support system, and the high workload all increase mental disorders [18]. In agreement with Alsharji [19], the severity of depression was correlated significantly with higher education levels which can be explained due to increases awareness of the current pandemic stressful news especially among the health care workers in the hospital.

Dyspnoea score mMRC was significantly correlated with the depression severity with *p* value < 0.01 (84.2% of moderate or severe depression group had grade 2 dyspnoea compared to 36.1% of no or mild depression group). With an aggravation of breathing discomfort, patients consider breathlessness as a threat associated with more depressive symptoms, which was confirmed by many studies and may induce secondary psychiatric responses. Therefore, dyspnoea may be associated with depressive symptoms in COVID-19 patients [20].

The aetiology of the psychiatric consequences of infection with coronavirus is likely to be multifactorial and might include the direct effects of viral infection (including brain infection), the degree of physiological compromise (e.g. hypoxia), cerebrovascular disease (including in the context of a procoagulant state), the immunological response, medical interventions, social isolation, the psychological impact of a novel severe and potentially fatal illness, concerns about infecting others, and stigma. The immune response in SARS-CoV-2 infection is of interest and there might be a hyperinflammatory state similar to that seen in haemophagocytic lymphohisticytosis in which there are increased concentrations of C-reactive protein, ferritin, and interleukin-6, although this state is likely to be short lived. The link between inflammation and depression is well described and might explain some of the psychiatric morbidity [21, 22].

In this work, 89.5% of moderate or severe depression group were using steroids compared to only 45.8% of no or mild depression group. Nearly the same was found by García et al., who found that with short course high-dose corticosteroid treatment (as occurred in COVID-19) may cause delirium and changes in mood (with a frequency of up to 52% of patients treated with more than 20 mg a day of prednisone during 3 months) [23].

Finally, COVID-19 infection even fear from infection and stigma of suspicion cause depression which was shown among sample of Egyptians causing a higher degree of dyspnoea and functional disability.

## Conclusions

Age, sex, and education level were important association factors with the prevalence of psychiatric sequelae in COVID-19 survivors. Higher educational level was associated with higher score of depression due to increased awareness of the current pandemic issue. Steroids’ use was proposed as a cause of depression since the majority of moderate or severe depression group were on steroids. Higher grades of dyspnoea were associated with higher probability of development of moderate or severe post-COVID depression. It is suggested that COVID-19 survivors should be assessed, to properly diagnose and treat any psychiatric conditions, to reduce the disease burden. Further work up is needed to investigate how the immune-inflammatory response is translated into psychiatric illness to improve our knowledge of these disorders.

## Data Availability

The data sets used during the current study are available from the corresponding author on reasonable request.

## Abbreviations

COVID-19: Coronavirus disease 2019
CoVs: Coronaviruses
MERS: Middle East respiratory syndrome
SARS: Severe acute respiratory syndrome
CRP: C reactive protein
HCWs: Health care workers
HRCT: High resolution computed tomography
mMRC: Modified Medical Research Council
BDI: Beck depression inventory
